# Fiber-specific micro- and macroscopic white matter alterations in progressive supranuclear palsy and corticobasal syndrome

**DOI:** 10.1038/s41531-023-00565-2

**Published:** 2023-08-17

**Authors:** Wataru Uchida, Koji Kamagata, Christina Andica, Kaito Takabayashi, Yuya Saito, Mana Owaki, Shohei Fujita, Akifumi Hagiwara, Akihiko Wada, Toshiaki Akashi, Katsuhiro Sano, Masaaki Hori, Shigeki Aoki

**Affiliations:** 1 Department of Radiology, Juntendo University Graduate School of Medicine, Bunkyo-ku, Tokyo 113-8421 Japan; 2 Faculty of Health Data Science, Juntendo University, Urayasu, Chiba 279-0013 Japan; 3 Department of Radiological Sciences, Graduate School of Human Health Sciences, Tokyo Metropolitan University, Arakawa-ku, Tokyo 116-8551 Japan; 4 Department of Radiology, Toho University Omori Medical Center, Ota-ku, Tokyo 143-8541 Japan

**Keywords:** Diagnostic markers, Predictive markers, Neurodegenerative diseases

## Abstract

Progressive supranuclear palsy (PSP) and corticobasal syndrome (CBS) are characterized by progressive white matter (WM) alterations associated with the prion-like spreading of four-repeat tau, which has been pathologically confirmed. It has been challenging to monitor the WM degeneration patterns underlying the clinical deficits in vivo. Here, a fiber-specific fiber density and fiber cross-section, and their combined measure estimated using fixel-based analysis (FBA), were cross-sectionally and longitudinally assessed in PSP (*n* = 20), CBS (*n* = 17), and healthy controls (*n* = 20). FBA indicated disease-specific progression patterns of fiber density loss and subsequent bundle atrophy consistent with the tau propagation patterns previously suggested in a histopathological study. This consistency suggests the new insight that FBA can monitor the progressive tau-related WM changes in vivo. Furthermore, fixel-wise metrics indicated strong correlations with motor and cognitive dysfunction and the classifiability of highly overlapping diseases. Our findings might also provide a tool to monitor clinical decline and classify both diseases.

## Introduction

Progressive supranuclear palsy (PSP) and corticobasal degeneration (CBD) are four-repeat (4R) tauopathies characterized by the accumulation of 4R-tau in the neurons and glia^1,2^, with general absence of beta-amyloid deposits, unlike in Alzheimer’s disease^3^. PSP is clinically associated with progressive gait failure, vertical gaze palsy, dysarthria, and dysphagia^4,5^. Moreover, PSP is characterized by vulnerability to tau toxicity and aggregation, not only in the midbrain and superior cerebellar peduncle (SCP), which is known as a hallmark, but also in the tracts under the premotor and motor cortices^6,7^. Although CBD has a variety of phenotypes, the most prototypical clinical phenotype is corticobasal syndrome (CBS), which presents with cognitive deficits and extrapyramidal motor features such as asymmetric limb rigidity, dystonia, myoclonus, and apraxia and cortical sensory deficit^8,9^. Previous studies suggested that at least the motor cortex and subcortical structures are commonly involved in diverse diseases underlying the CBS^10,11^.

Both PSP and CBS are characterized by selective white matter (WM) degeneration due to the patterns of prion-like spread of tau pathology through the axons^12^. Changes in WM topology and connectivity are driven by the patterns of the spread of tau aggregation in neurodegenerative tauopathy^13^ that cause dysfunction of axons and glial cells^14–16^. Given this feature and the effect of 4R-tau distributions on clinical symptoms, understanding the micro- and macroscopic structures of WM that correspond to tau depositions in neurodegenerative tauopathies could be useful in the diagnosis and follow-up of PSP and CBS. However, the mechanisms underlying the disturbances in the integrity of WM that lead to the progressions of clinical dysfunction are not fully elucidated; thus, hindering the development of therapies that could provide adequate functional improvement in both diseases. Furthermore, due to a high degree of overlapping symptomatology between the two diseases, differentiating PSP and CBS is often challenging using current in vivo assessments, especially in the early stages of the disease. These facts emphasize the need for objective biomarkers, including a diagnostic biomarker to accurately classify PSP and CBS, the monitoring biomarker to capture patterns of disease progression, the predictive biomarker to estimate intervention effects, and guide disease-modifying therapies^17^.

Previous WM structural assessment studies in PSP and CBS have focused primarily on morphological atrophy based on structural MRI^18,19^. Recently, however, there has been an increased focus on the importance of assessing microstructural degeneration that typically precedes macrostructural atrophy using diffusion tensor imaging (DTI)^20–22^. Most of these are voxel-based analyses, which determine the average value of a quantitative index within a single voxel. Despite the evidence for different WM alteration patterns in PSP and CBS according to voxel-based analysis, sufficient knowledge has not been accumulated to establish WM alterations as an in vivo biomarker, likely due to difficulties with interpretation derived from the partial volume effect that arises from the crossing fiber population (up to 90% of WM voxels)^23^. Considering this scenario, there are high expectations for fixel-based analysis (FBA), which can model complex fiber geometry in multiple directions within a single voxel and evaluate both micro- and macroscopic neural structures within a fiber-specific grid (i.e., “fixel”)^18,24^. FBA can estimate fixel-wise parameters, including fiber density (FD; a microscopic parameter corresponding to the axonal density), fiber-bundle cross-sections (FC; a macroscopic parameter of fiber-bundle cross-sections), and a combination of both denaturation processes (fiber density and bundle cross-section; FDC; a micro- and macroscopic parameter that is sensitive, especially in the fixel grids, wherein differences in both FD and FC appear)^18^.

Recently, a few studies adopted FBA to assess WM integrity in PSP and CBS. A previous study found both micro- and macroscopic WM changes in PSP, along the corpus callosum (CC) and descending fibers from the motor cortex, more conspicuous than Parkinson’s disease^25^. Another study reported a higher degree of FD reduction along the CC and bundle atrophy along the projection fibers from the motor cortex in CBS than in Parkinson’s disease. They also emphasized the contribution of the dentatorubrothalamic tract (DRTT), including the SCP in CBS^26^. However, there has been no FBA study that directly compared WM integrity in PSP and CBS or evaluated the association between WM changes and actual clinical dysfunction using a longitudinal approach. Insights that contribute to the development of a useful biomarker and help to fully understand WM structural changes underlying rapid progressive motor and cognitive dysfunctions in PSP and CBS have been limited. Thus, it is important to evaluate the structural changes of WM in PSP and CBS cross-sectionally and longitudinally, including more detailed morphological information and changes in clinical indices.

Thus, in the current study, we aimed to investigate if FBA can serve as an imaging biomarker defined by the FDA and the National Institutes of Health Biomarkers, EndpointS, and other Tools (BEST)^17^ including (1) the monitoring biomarker to grasp the progressive WM degeneration patterns in PSP and CBS that underlie these clinical dysfunctions from micro- and macroscope perspectives, (2) the diagnostic biomarker to accurately classify patients with PSP and CBS in vivo, and (3) the prognostic biomarker to predict future dysfunctions in the early stages.

## Results

### Participant’s demographic and clinical information

Table 1 shows the demographic information and the longitudinal changes in the clinical parameters. Age and sex at baseline were matched statistically among the three groups (*P* < 0.05 in ANOVA for age and in χ2 test for sex). The disease duration at baseline was also not significantly different between patient groups. Significant group differences at baseline between patients and HC were observed in the PSP Rating Scale (PSPRS) Total, Mini-Mental State Examination (MMSE), Montreal Cognitive Assessment (MoCA), and the Schwab and England Actives of Daily Living (SEADL) as shown in Table 1. Meanwhile, the PSPRS subscores of “Bulbar” and “Gait and midline” were significantly lower in CBS compared with that in PSP, and the PSPRS subscores for limb motor were significantly lower in PSP than that in CBS.

**Table 1:** Participants’ demographics, clinical information, and magnetic resonance findings.

	HC (*N* = 20)	PSP (*N* = 20)		CBS (*N* = 17)		Cross-sectional group differences at baseline			Longitudinal changes		
	Baseline	Baseline	Follow-up	Baseline	Follow-up	PSP vs. HC	CBS vs. HC	PSP vs. CBS	PSP	CBS	PSP vs. CBS
**Age, year**	64.7 ± 6.1	69.0 ± 6.5	70.0 ± 6.5	65.6 ± 7.1	66.6 ± 7.1	n.s.	n.s.	n.s.	-		
**Sex, % male**	30.0%	55.0%	-	29.4%	-	n.s.	n.s.	n.s.			
**Disease duration, year**	-	4.4 ± 3.6	5.0 ± 3.6	4.5 ± 2.4	5.5 ± 2.4	n.s.	n.s.	n.s.	-	-	-
**PSPRS Total**	0.61 ± 1.42	30.4 ± 11.7	39.0 ± 12.8	28.3 ± 11.2	36.9 ± 15.7	**<0.001**	**<0.001**	n.s.	**0.005**	**0.042**	n.s.
PSPRS History	-	7.42 ± 2.78	8.17 ± 2.51	6.45 ± 2.87	7.73 ± 3.69	-	-	n.s.	n.s.	n.s	n.s.
PSPRS Mention	-	2.42 ± 1.55	3.42 ± 3.33	3.09 ± 1.83	3.82 ± 3.16	-	-	n.s.	n.s.	n.s	n.s.
PSPRS Bulbar	-	2.08 ± 1.38	2.67 ± 1.43	1.55 ± 1.08	2.09 ± 1.78	-	-	**0.028**	n.s.	n.s	n.s.
PSPRS Oculomotor	-	5.42 ± 3.30	7.42 ± 3.80	2.00 ± 2.41	4.45 ± 3.82	-	-	**<0.001**	**0.012**	**0.011**	n.s.
PSPRS Limb motor	-	4.50 ± 2.43	5.17 ± 2.79	7.09 ± 2.57	9.09 ± 3.96	-	-	**0.003**	n.s.	**0.035**	n.s.
PSPRS Gait and midline	-	8.58 ± 3.66	12.2 ± 3.72	8.18 ± 5.52	9.73 ± 5.74	-	-	n.s.	**0.003**	**0.031**	**0.046**
**UPDRS-**III **Total**	-	26.1 ± 13.6	34.1 ± 15.0	32.0 ± 14.2	41.3 ± 17.4	-	-	n.s.	**0.004**	**0.049**	n.s.
**MMSE**	29.2 ± 1.0	27.1 ± 2.3	25.1 ± 2.5	22.9 ± 5.4	20.4 ± 8.2	**0.001**	**<0.001**	n.s.	**0.006**	n.s.	n.s.
**MoCA**	28.2 ± 1.6	22.7 ± 2.4	20.7 ± 3.0	17.4 ± 7.0	14.9 ± 7.5	**<0.001**	**<0.001**	n.s.	**0.049**	n.s.	n.s.
**Clinical Dementia Rating Box**	-	3.23 ± 1.86	3.93 ± 3.17	3.46 ± 2.27	5.50 ± 4.26	-	-	n.s.	n.s.	n.s.	n.s.
**Functional Activities Questionnaire**	-	14.6 ± 6.6	15.6 ± 5.6	11.8 ± 6.6	16.5 ± 7.7	-	-	n.s.	n.s.	n.s.	n.s.
**SEADL**	100	55.8 ± 25.6	48.3 ± 22.3	48.9 ± 20.3	32.2 ± 18.1	**<0.001**	**<0.001**	n.s.	n.s.	n.s	n.s.

Bold values denote statistical significance (*P* < 0.05). n.s. not significant.

After 1 year, we observed the motor and cognitive decline in PSP and CBS. Specifically, in PSP, we found significantly increased PSPRS Total and motor scores, including PSPRS subscores of “Oculomotor,” “Limb motor,” and “Gait and midline,” and Unified Parkinson’s Disease Rating Scale part 3 (UPDRS-III). PSP also showed a significant cognitive decline in MoCA over 1 year. On the other hand, in CBS patients, per the total PSPRS score and motor scores of PSPRS subscores for “Oculomotor” and “Gait and midline,” UPDRS-III scores were significantly worsened longitudinally. The cognitive score of MMSE also significantly decreased longitudinally. In the comparisons of changes in clinical deficits, the degree of changes in the PSPRS subscores for “Gait and midline” was significantly severe in PSP compared with that in CBS.

### Whole-brain FBA

**Fig. 1:**
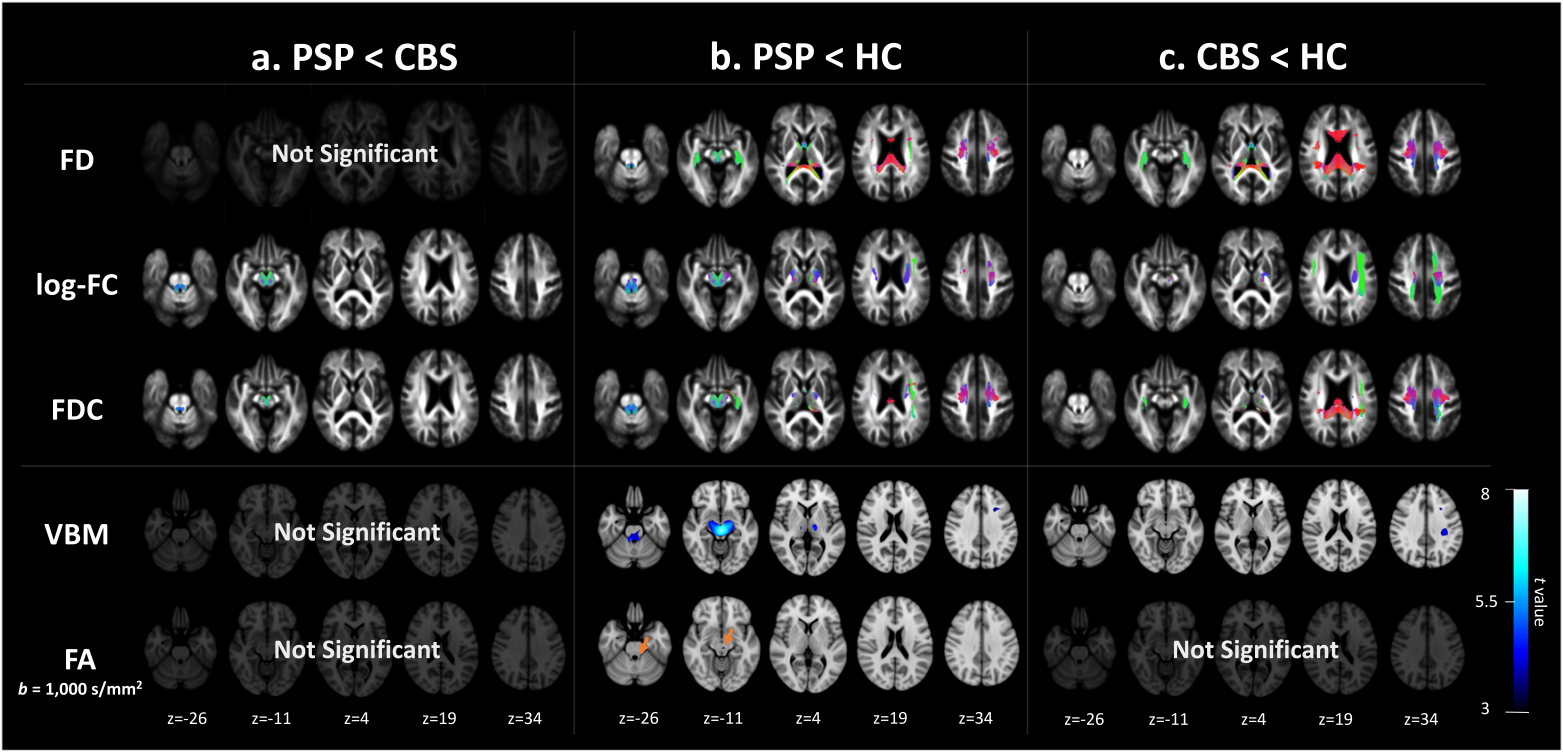
Cross-sectional group differences in the fixel-based metrics. Column **a** shows the significant decline in PSP compared with CBS. Significantly lower indices compared with HC in PSP are shown in column (**b**), and those in CBS are shown in column (**c**). In the upper panel, the streamlines cropped from template tractograms passing significant fixels are shown as colored by direction: red, left-right; green, anterior-posterior; and blue, inferior-superior, as the results of fixel-based analysis (family-wise error-corrected *P* < 0.05). The lower panel shows the results of voxel-based analysis, including voxel-based morphometry (VBM) and voxel-based quantification comprising the fractional anisotropy. The significant voxels (family-wise error-corrected *P* < 0.05) were scaled by the *t*-values, ranging from 3 to 8.

At baseline comparisons, all FBA metrics were significantly changed (family-wise error-corrected *P*-value < 0.05) in both PSP and CBS compared with HCs. In PSP, we found the lower FD, log-FC, and FDC, specifically in the SCP, and in WM tracts, including the corticospinal tract (CST), superior longitudinal fasciculus (SLF), and body of the CC, corresponding to the motor and frontal areas (Fig. 1b). The fixels in the SCP and the midbrain were also shown as a significantly reduced log-FC and FDC in PSP compared with CBS (Fig. 1a). The significant fixels in log-FC and FDC were located across the midline, coursing toward the ventrolateral thalamic nucleus via the red nucleus, constructing the decussation. Contrarily, in CBS patients (Fig. 1c), widespread FD and log-FC changes were observed compared with HCs. There was significantly decreased FD in the fornix, genu, body, and splenium of the CC and log-FC in the CST, SLF, cingulum, and body of the CC compared with HCs. The reduced FDC generally overlapped on the tracts that were significantly changed in FD or log-FC.

**Fig. 2:**
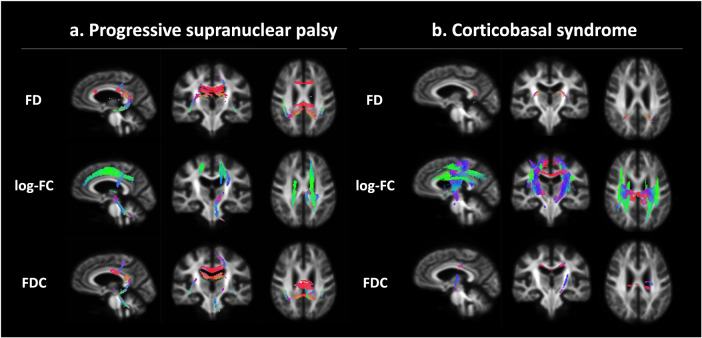
Longitudinal changes in the fixel-based metrics over 1 year in patients with progressive supranuclear palsy (PSP) and corticobasal syndrome (CBS). The streamlines that were cropped from template tractograms passing significant fixels (family-wise error-corrected *P* < 0.05) are shown as colored by direction: red, left-right; green, anterior-posterior; and blue, inferior-superior, in the longitudinal fixel-based analysis of PSP shown in column (**a**) and CBS shown in column (**b**).

Longitudinal FBA (Fig. 2) detected significantly decreased log-FC in projection fibers corresponding to the motor cortex and in the SLF, accompanied by further changes in the SCP and the midbrain. In addition, the FD loss was observed in relatively widespread association fibers, including the genu, body, and splenium of CC. Despite the different WM geometry states of each WM region in PSP, the longitudinally reduced log-FC was dominant in CBS changes. Significant changes in log-FC were limited mainly in the supratentorial WM, including the projection fibers under the motor cortex and SLF. In both diseases, the significant changes in FDC were generally overlapped with the changes in FD or log-FC.

### Voxel-based analysis

Figure 1 also shows the results of whole-brain voxel-based analysis at baseline. Voxel-based morphometry (VBM) analysis detected significantly atrophied WM in the midbrain and SCP in PSP versus that in the HCs as well as the WM under the motor cortex in CBS versus that in the HCs. Further, voxel-based quantification (VBQ) detected significantly decreased fractional anisotropy (FA) in the bilateral SCP and midbrain in a comparison between PSP and HCs. CBS revealed no significant changes. Both VBM and VBQ indicated no significant changes between PSP and CBS.

No significant changes in voxel-wise metrics were shown in both PSP and CBS over a 1-year period.

### Fixel-wise tract-specific analysis

**Fig. 3:**
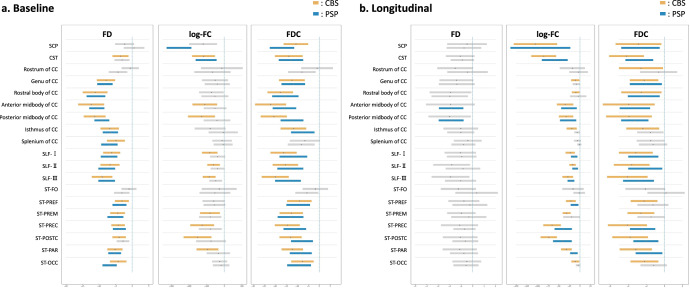
Cross-sectional and longitudinal fixel-wise tract-specific analysis in the progressive supranuclear palsy (PSP) and corticobasal syndrome (CBS). **a** The cross-sectional mean percent changes and 95% confidence intervals in the fiber density (FD), log-transformed fiber cross-section (log-FC), and fiber density and cross-section (FDC) are displayed for PSP and CBS compared to those of healthy controls. The significantly decreased tracts are colored with blue for PSP and orange for CBS, and the tracts that did not show significant changes are displayed as gray bars. **b** The longitudinal mean percent changes and 95% confidence intervals in the FD, log-FC, and FDC are displayed compared to the baseline metrics in PSP and CBS. The significantly altered tracts over 1 year are colored, and the no significant tracts are shown as gray bars. SCP Superior cerebellar peduncle, CST corticospinal tract, CC corpus callosum, SLF-I superior longitudinal fascicle I, SLF-II superior longitudinal fascicle II, SLF-III superior longitudinal fascicle III, ST-FO striato-fronto-orbital, ST-PREF striato-prefrontal, ST-PREM striato-premotor, ST-PREC striato-precentral, ST-POSTC striato-postcentral, ST-PAR striato-parietal, ST-OCC striato-occipital.

In the baseline comparisons (Fig. 3a), microscopic changes in WM were consistent with the whole-brain FBA and were observed in the CC and striato-cortical pathways relating to the frontal, parietal, and occipital lobes and SLF in both patient groups according to FD. In addition, decreased FD along the CST was observed only in CBS. On the other hand, macroscope WM changes (i.e., decreased log-FC) were observed mainly in CBS along the CST, SLF, and parts of the CC and striato-cortical pathways, which were comparably more widespread than the whole-brain FBA. In PSP, the lower log-FC was detected only in the SCP and CST. The significantly lower FDC had similar patterns in the FD and log-FC results, and the magnitude of changes in FD, log-FC, and FDC were consistently noticeable in the WM tracts corresponding to the motor cortex.

Longitudinal FDC change patterns were similar in both diseases (Fig. 3b), as well as the results of whole-brain analysis. Significantly decreased log-FC was found in the subcortical WM, corresponding to the motor cortex in the PSP and relatively extensive cortex in CBS and the SCP in both diseases. A particularly high degree of macroscopic degeneration over 1 year was shown in the SCP and commissural and projection WM under the motor cortex, which were common trends in PSP and CBS. Decreased FD was specifically shown in the association fibers corresponding to the motor cortex in PSP. Significantly lower FDC was roughly congruent with the log-FC results and was more sensitive than the whole-brain results in CBS in contrast to consistency in the PSP. Longitudinal FBA parameter changes were the greatest in the WM tracts under the motor cortex in PSP and CBS, which was also indicated in the baseline comparisons and whole-brain analysis.

### A receiver operating characteristic analysis and a linear discriminant analysis

To verify the possibility that fixel-wise metrics can accurately diagnose PSP and CBS, the log-FC and FDC in the part of SCP that significantly differed between the diseases were utilized for a linear discriminant analysis using leave-one-out cross-validation.

The FDC yielded an area under the curve (AUC) of 0.82 (with a sensitivity of 85% and specificity of 71%), while the log-FC yielded an AUC of 0.80 (with a sensitivity of 75% and specificity of 65%) when examining the specific SCP region that exhibited a significant difference between PSP and CBS. In contrast, the diagnostic performance decreased, with an AUC of 0.60 (sensitivity: 70%, specificity: 35%) for FDC and an AUC of 0.70 (sensitivity: 80%, specificity: 65%) for log-FC when evaluating the entire SCP. In a receiver operating characteristic analysis, the classical MRI results were: sensitivity of 55% and specificity of 71% in the hummingbirds sign; and sensitivity of 71% and specificity of 75% in the asymmetrical frontoparietal atrophy. The expert’s diagnosis indicated high reliability (absolute inter-rater agreement, 89% [κ = 0.78, *P* < 0.001] in the hummingbirds sign, and 92% [κ = 0.84, *P* < 0.001] in asymmetrical frontoparietal atrophy).

### Correlation analysis

**Fig. 4:**
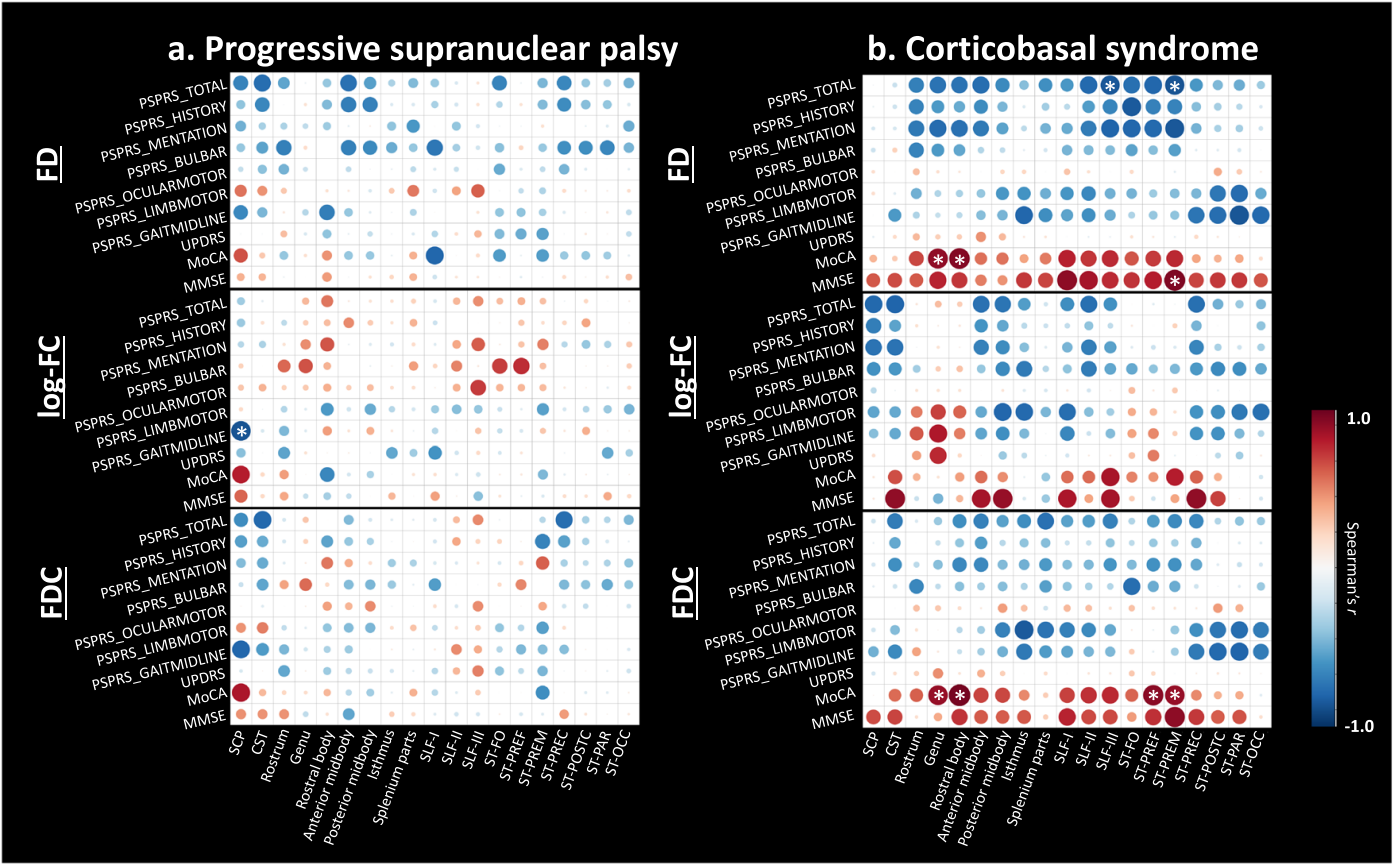
Partial correlation between the longitudinal changes of the fixel-wise parameters and clinical indices. The partial correlation (Spearman’s *r*) adjusted for age, sex, and disease duration in progressive supranuclear palsy (**a**) and corticobasal syndrome (**b**) are illustrated as heatmaps, which are colored red (positive; Spearman’s *r* range, 0 to 1) to blue (negative, Spearman’s *r* range, −1 to 0). The diameter of the circles in each grid are scaled with the absolute Spearman’s r, which was set 1 as the maximum and 0 as the minimum. The asterisks on the circles represent a significant correlation (false-discovery ratio-corrected *P* < 0.05). SCP Superior cerebellar peduncle, CST corticospinal tract, CC corpus callosum, SLF-I superior longitudinal fascicle I, SLF-II superior longitudinal fascicle II, SLF-III superior longitudinal fascicle III, ST-FO striato-fronto-orbital, ST-PREF striato-prefrontal, ST-PREM striato-premotor, ST-PREC striato-precentral, ST-POSTC striato-postcentral, ST-PAR striato-parietal, ST-OCC striato-occipital, PSPRS_TOTAL Total progressive supranuclear palsy rating scale, PSPRS_HISTORY PSPRS subscores of “History,” PSPRS_MENTATION “Mentation,” PSPRS_BULBAR “Bulbar,” PSPRS_OCULARMOTOR “Oculomotor,” PSPRS_LIMBMOTOR “Limb motor,” PSPRS_GAITMIDLINE “Gait and midline,” UPDRS Unified Parkinson’s Disease Rating Scale part 3, MMSE Mini-Mental State Examination, MoCA Montreal Cognitive Assessment.

Figure 4 shows that the changes in the fixel-wise indices along some tracts were associated with the actual changes in the clinical scores. Significant correlations were observed between the degree of decrease in log-FC along the SCP (*r* = −0.87, the false-discovery rate [FDR]-corrected *P* = 0.023) and the degree of severity over 1 year for subscores of “Gait and midline” in the PSP. In CBS, the degree of decrease in FD and FDC were significantly correlated with the deterioration of PSPRS Total, MoCA, and MMSE in the WM tracts, as follows: genu of CC (FD-MoCA, *r* = 0.89, FDR-corrected *P* = 0.0031; FDC-MoCA, *r* = 0.87, FDR-corrected *P* = 0.0048), rostral body of CC (FD-MoCA, *r* = 0.92, FDR-corrected *P* = 0.0012; FDC-MoCA, *r* = 0.96, FDR-corrected *P* = 0.0012), SLF-III (FD-PSPRS Total, *r* = −0.85, FDR-corrected *P* = 0.0039), striato-prefrontal pathway (FDC-MoCA, *r* = 0.90, FDR-corrected *P* = 0.0021), and striato-premotor pathway (FD-PSPRS Total, *r* = −0.86, FDR-corrected *P* = 0.0028; FD-MMSE, *r* = 0.93, FDR-corrected *P* = 0.0022; FDC-MoCA, *r* = 0.87, FDR-corrected *P* = 0.0048).

### Linear regression analysis

**Fig. 5:**
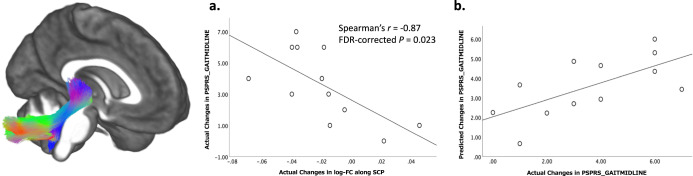
The association between longitudinal changes in the clinical index and the bundle cross-section. **a** The scatter plot represents the association between actual changes of a progressive supranuclear palsy rating scale subscore for “gait and midline” (PSPRS_GAITMIDLDINE) and actual changes in log-transformed fiber cross-sections (log-FC) along the superior cerebellar peduncle (SCP). **b** The scatter plot represents the association between actual changes in PSPRS_GAITMIDLINE and the predicted changes of PSPRS_GAITMIDLINE derived from a linear regression analysis.

A forward selection in the linear regression model was used to test the possibility that fixel-wise metrics could predict future dysfunction in PSP and CBS (Fig. 5). The mean log-FC along SCP in PSP significantly predicted changes in the subscore “Gait and midline” (*F* = 7.67, *P* = 0.02 in ANOVA; *B* = 14.47, *P* = 0.02), and the variables estimated in the bootstrap procedure were significantly predictive (*B* = 14.47, *P* = 0.005, confidence interval = 7.10–22.92, bias = 0.048). In contrast, there were no significant measures along the tracts that were associated with the clinical measures in CBS. The ANOVA results were: genu of CC (FD-MoCA, *F* = 1.23, *P* = 0.3; FDC-MoCA, *F* = 1.59, *P* = 0.24), rostral body of CC (FD-MoCA, *F* = 1.33, *P* = 0.28; FDC-MoCA, *F* = 2.03, *P* = 0.19), SLF-III (FD-PSPRS Total, *F* = 4.08, *P* = 0.74), striato-prefrontal pathway (FDC-MoCA, *F* = 0.83, *P* = 0.39), and striato-premotor pathway (FD-PSPRS Total, *F* = 2.06, *P* = 0.19; FD-MMSE, *F* = 3.38, *P* = 0.11; FDC-MoCA, *F* = 0.86, *P* = 0.38).

## Discussion

We performed cross-sectional and longitudinal FBAs to provide insights into the progressive WM changes in PSP and CBS. PSP patients had lower FD and FDC along the SLF and widespread CC and projection fibers at baseline. Specifically, lower log-FC was found in the SCP and CST. In contrast, lower log-FC along WM under the motor cortex and lower FD and FDC were widely observed in the WM in CBS. These findings were consistent with the distribution patterns of 4R-tau recognized in PSP and CBS^3,7,11,27,28^. Next, a direct comparison between PSP and CBS indicated lower log-FC and FDC only in the SCP and midbrain, which accurately classified patients with PSP and CBS. We also demonstrated progressive WM degeneration in PSP and CBS after 1 year. Especially in PSP, the extent of atrophy in the SCP at baseline was shown to be a predictor of future dysfunction of stability. These findings suggest the possibility that FBA can monitor disease progression and classify patients with PSP and CBS.

FBA can assess both fiber-specific FD corresponding to the intra-axonal volume and fiber-specific FC, indicating macroscopic bundle atrophy subsequently occurred^29^. In PSP at baseline, the lower fixel-wise metrics were especially localized on the SCP and midbrain, and WM under the frontal and motor area that the previous histopathological studies reported tau depositions^3,7,27^. Prominent WM degeneration might reflect axonal degeneration and neuronal loss from neurofibrillary and globose tangles affected by hyperphosphorylated tau in the frontal and motor cortices^3,7^, in addition to SCP neuronal loss, which is a hallmark of PSP pathology^30^. CBS had severe bundles atrophy which were observed in extensive WM areas corresponding to the frontal and parietal lobes, including the motor cortex. The distribution of significant fixels was consistent with the distribution of prominent tau in CBD pathology^11,27,28^, which is the most frequent phenotype^31,32^. Particularly severe bundle atrophy in the WM descending to the motor cortex might support the hypothesis of the involvement of 4R-tau accumulation in the motor cortex at CBS symptom onset^27^. This trend was demonstrated in previous studies indicating atrophy of WM under the motor cortex in CBD, which clinically presented with CBS symptoms despite variable phenotypes in CBD^11,28^. This might indicate a disease mechanism of CBS; however, verification requires further studies with case-confirmed pathological diagnoses.

The longitudinal FBA indicated further WM atrophy along the SCP and CST and axonal degeneration and bundle atrophy along the WM under the motor cortex in PSP. CBS also showed consistent continuous changes in the WM under the cerebral cortex and even the SCP as a part of the midbrain. Recent pathological studies suggested the continuous spreading of tau propagation in CBD and PSP (Supplementary Fig. 1a)^33^. In PSP, neurofibrillary tangles were observed in the following: (1) subthalamic nucleus, globus pallidus, and substantia nigra; (2) striatum, brainstem, and motor cortex; (3) dentate nucleus, and amygdala; (4) frontal lobe; (5) parietal, temporal, and occipital lobes^3,7,34^. In contrast, CBD showed prominent tau distribution in the basal ganglia, including the striatum, in the early stages, primary motor and frontal cortex in the mid-stages, and parietal, temporal, and midbrain nucleus in the later stages. Although these schemes in cortical lobes were largely based on tau distribution in the cortex, previous neuropathological and biochemical studies demonstrated similar patterns of tau load on WM in the corresponding lobes^27,28,35,36^. In summary, the WM in PSP and CBS is continuously affected by tau pathology corresponding to the scheme of each disease. A previous longitudinal FBA study that evaluated relatively early-stage PSP patients indicated the FD loss in the WM under the motor cortex, which was the consistent region with our findings^37^. The reduced log-FC in the WM under the motor cortex might be attributable to differences in methodology, disease duration, and patients’ PSP severity. Although further verification is needed, this inconsistency might reflect the FBA’s ability to capture the bundle atrophy that occurred following FD reduction through the early involvement of the WM under the motor cortex. Additionally, tract-specific analyses indicated that the longitudinal log-FC changes along the SCP not only in PSP but also in CBS. This feature should be interpreted with caution due to the discrepancy in results across approaches; however, given the relatively late involvement of tau in the above scheme^27,28,35,36^, this feature might indicate the SCP atrophy in relatively late-stage CBS. Thus, our cross-sectional and longitudinal findings may indicate that the progressive axonal loss and the subsequent bundle atrophy are associated with tau toxicity and disease severity.

**Fig. 6:**
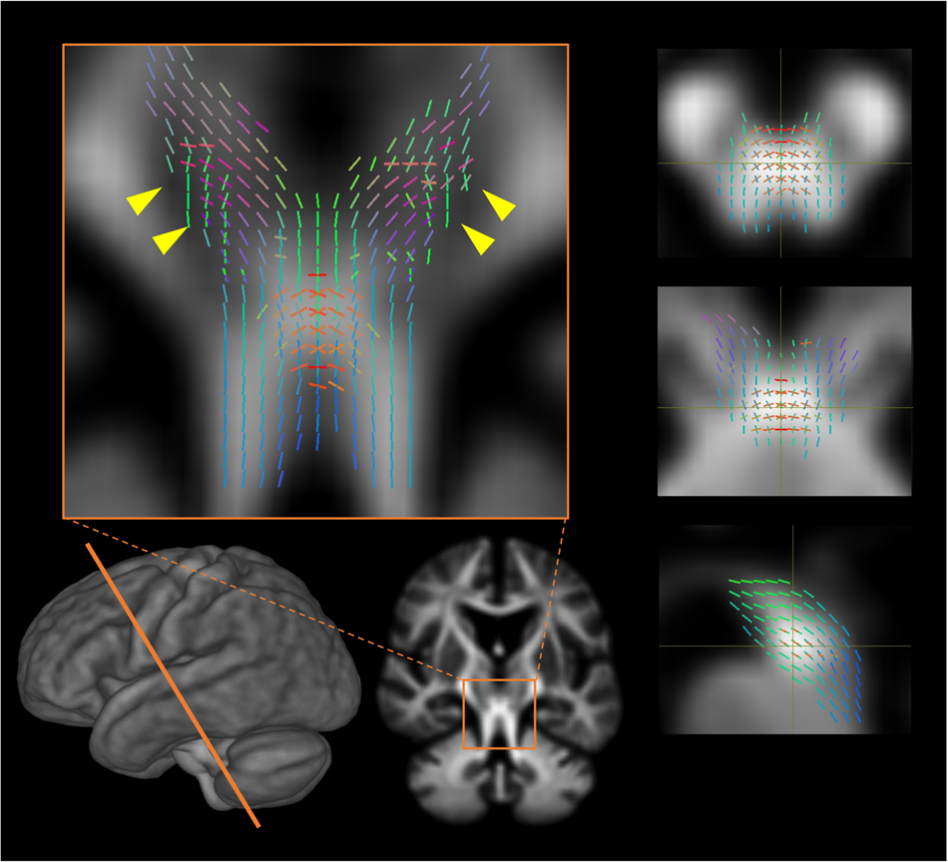
Decussation of the superior cerebral peduncle construction with the significant fixels. The shown fixels are significantly decreased in the log-transformed fiber cross-section in progressive supranuclear palsy compared to that in corticobasal syndromes (family-wise error-corrected *P* < 0.05) colored by direction: red, left-right; green, anterior-posterior; and blue, inferior-superior. The significant fixels construct the decussation of the superior cerebellar peduncle through the contralateral red nucleus (yellow arrowheads).

Despite previous neuroimaging results showing atrophy in the SCP based on VBM^22,38^, we observed both micro- and macro-WM degeneration along bilateral DRTTs as confirmed by constructing a fixel-decussation of the SCP, suggesting the superiority of FBA in delineating crossing fibers (Fig. 6)^18,24^. Incorporating recent work that demonstrated non-decussating DRTT and its different functions and endpoints^39,40^, our results also provide new insights into the involvement of decussating DRTT in PSP pathology. It is worth noting that bundle atrophy in the SCP progressed over 1 year in association with the “Gait and midline” score. PSP is characterized by the 4R-tau burden in neurons and oligodendrocytes with axonal loss^34^ and demyelination indirectly associated with axonal loss^41^ in the earliest stage. Pathology in the DRTT is commonly found^4,42^ and associated with frequent falls and postural instability^2,30^. In summary, our results might reflect that bundle atrophy occurs following axonal degeneration, which is indirectly associated with neuronal and oligodendrocyte pathology. Remarkably, the DRTT reduction in PSP was related to progressive gait dysfunction and postural instability in PSP and could better classify both diseases. The consistently high performance of log-FC across various measurements indicates its robustness in classifying mid- to late-stage diseases. These findings are attributed to fiber-specific atrophy in CBS, which manifests in the later stages. Therefore, FBA can differentiate WM integrity based on the disease stage or severity of PSP and CBS, but further validation is necessary. Although the hummingbirds sign and asymmetrical frontoparietal atrophy have been generally recognized as typical imaging features, they are not specific. Considering tau vulnerability in the midbrain tegmentum in PSP^34^ and a preserved brainstem structure in mid- to late-stage CBS phenotypes^10^, the fixel-wise metrics along brainstem and SCP might provide a potential target for understanding the disease mechanisms in the context of differentiating PSP and CBS.

There is an increasing demand for non-invasive and expedient biomarkers that predict clinical symptoms in PSP and CBS. Bundle atrophy along the SCP in PSP was specifically correlated with the PSPRS subscores of “Gait and midline,” and log-FC at baseline could precisely predict subsequent deterioration. Previous studies using DTI reported this feature as well^43,44^ and suggested the role of the SCP in gait and postural stability^45^. Postmortem studies also suggested degeneration of red nuclei and other nuclei bordered by the SCP relates to dysfunctions of gait initiation and postural instability^46^. Thus, the current results emphasized the contribution of the SCP as a prognostic biomarker for gait impairment and instability in PSP. On the other hand, the strong correlation between PSPRS Total scores and cognitive scores, and the structural alterations in WM, corresponding to primary and sensory motor cortices, agreed with prominent tau pathology observed from mid-staged CBD^28^. The current results might derive from various clinical dysfunctions such as mentation, limb, oculomotor and bulbar dysfunctions, and stability in CBS. Furthermore, CBS showed progressive cognitive decline had a strong association with longitudinal changes in FD along the WM of the frontal and motor cortices. In line with previous findings on early involvement of the frontoparietal network related to cognitive dysfunction in CBD^47,48^, our results might provide new insights into the microstructural WM changes underlying cognitive impairment in CBS. Notably, one longitudinal study using DTI reported the correlation between its rates of change in the motor subcortical WM and the clinical deficits mainly in limb and ocular motor neurons, which was contrary to our findings^49^. This inconsistency might be accounted for through the different disease durations, scan intervals, and analytic approaches. Further studies are needed to solve the discrepancy.

FBA is methodologically useful in that it can assess both progressive fiber structural changes and crossing fibers, especially in a voxel that has multi-directional fibers^29^. Certainly, in addition to the detectability of fixel-decussation of SCP, the significantly changed fixels (i.e., reduced FD or log-FC) completely represented each tract beyond contamination by other voxels where the CC and projection fibers intersect, which showed the superiority over voxel-wise DTI (Supplementary Fig. 2). Focusing on this region, which corresponds to subcortical WM under the motor cortex, bundle atrophy of projection fibers and reduced FD in the CC were observed in PSP, whereas bundle atrophy and further fiber damage already occurred as reduced FD in the CC in CBS at baseline (Fig. 3). These features were also shown along the striato-motor pathways as well as CC projections in both hemispheres (Supplementary Fig. 1). Incorporating the scenario that FBA can assess FD and subsequent bundle atrophy, our findings reflect WM alteration patterns as a result of early tau vulnerability of the motor cortex and striatum and subsequent patterns of tau propagation to other affected cortical areas in CBS^27,28,35,36^ and PSP’s prominent tau pathology in the motor cortex, which occurs later but is the earliest affected compared to other cortical areas^3,7,34^. These progressive WM degeneration patterns based on FBA methodology were also observed in longitudinal WM alterations as the most prominent WM changes along motor cortex tracts. In summary, the current study showed abnormal CCs with disease progression and differentiated between PSP and CBS, suggesting that the striato-cortical pathway can be a potential target for an imaging biomarker.

There are some limitations that should be considered. One is the lack of pathologic confirmation of diagnoses for all patients in our cohort. CBS has been reported to manifest in diverse diseases such as CBD, PSP, and Alzheimer’s disease^50^. Our results of the frontal and parietal subcortical WM atrophy in CBS have previously been reported^19,26^; however, VBQ analyses detected no significant changes in CBS, which might be influenced by the existence of diverse diseases underlying CBS. Certainly, exploratory DTI studies showed significantly lower FA in inconsistent WM^22,26,49,51,52^. Although it was difficult to validate due to the small sample size, our findings should be validated via postmortem confirmation or CBD selection with available biomarkers. Previous reports suggest the use of amyloid-beta^53^ and tau positron emission tomography^54^, total phosphorylated tau in CSF^55^, and the Magnetic Resonance Parkinsonism Index^56^ as biomarkers. The lack of validation using an independent test cohort should also be considered, while the generalizability of FBA is required to establish the monitoring, diagnostic, and prognostic biomarkers^17^. Future studies with adequate validation need to prove FBA to be a strong biomarker. We did not consider typical left-right differences in CBS because of the lack of clinical laterality information in the 4R Tauopathy Neuroimaging Initiative (4RTNI) database. This may have resulted in reduced sensitivity to detect neurodegeneration underlying clinical disabilities. Nevertheless, our results indicated prominent WM alterations might produce clinical impairments, which is consistent with previous histopathological findings. In this regard, the lack of histopathological properties underlying the fixel-wise metrics should be noted. Technically, it should also be noted that single-shell diffusion-weighted imaging (DWI) data (*b* = 2000 s/mm^2^) were obtained from 4RTNI and Neuroimaging Initiative for Frontotemporal Lobar Degeneration (FTLDNI) databases. The single-shell three-tissue constrained spherical deconvolution (SS3T-CSD) algorithm can model three-tissue compartments to address the partial volume effect by the gray matter and CSF contamination, and its accuracy is reportedly comparable to multi-shell acquisition^57^. However, although the deliberately chosen relatively high *b*-values from the databases to consider the SS3T-CSD algorithm was optimized for high *b*-values, higher *b*-value data or multi-shell data might have the ability to estimate the more robust FBA metrics^58,59^. Furthermore, the absence of the susceptibility-induced distortion correction due to the lack of reverse-phase encoded images in databases might have induced a greater variance to estimate the FOD model^60^.

We performed FBA for PSP and CBS, and FBA captured the loss of FD and subsequent bundle atrophy, which was congruent with the proposed tau propagation. This finding suggests a novel insight that FBA can monitor the progressive tau-related WM changes in vivo. Additionally, fixel-wise metrics indicated the correlation between motor and cognitive dysfunction in both diseases and the classifiability of these diseases. These findings provide the FBA’s potential ability to be a useful in vivo biomarker to monitor the clinical decline and to distinguish PSP and CBS that highly overlapping. Our results may also help to understand the mechanisms of WM degeneration underlying the disease progression. Future studies are needed to validate the association between changes in fixel-wise metrics and the underlying histopathology in both diseases.

## Methods

### Participants

CBS and PSP data were collected from the 4RTNI, and HC data were obtained from the FTLDNI. Ethical approval was not required because this study involves only participants from the above databases previously collected and fully anonymized. The original data collection involved obtaining written informed consent from all participants in this study, and the protocol was approved by the institutional review board at all participating sites. A previous study revealed improved accuracy and specificity of apparent fiber density using higher *b*-values and single-shell data, as opposed to multi-shell data that includes lower *b*-values^59^. Therefore, the present study incorporated study participants having diffusion-weighted images with a *b*-value of 2000 s/mm^2^ as part of the inclusion criteria. We only included participants with baseline and 1-year (range, 10–14 months) follow-up DWI data to facilitate a longitudinal investigation. Our dataset excluded patients with a history of significant psychiatric or neurological disorders other than that associated with PSP or CBS. All HCs were confirmed to have normal cognition with an MMSE score of ≥27. Eventually, our dataset included 57 subjects consisting of 20 PSP and 17 CBS patients and 20 HCs. Patients with PSP were diagnosed with the National Institute of Neurological Disorders the Stroke/Society for PSP criteria for PSP-Richardson syndrome^61^. All patients with CBS met the requirements for the Armstrong criteria for CBS-CBD subtypes^9^. Patients underwent neurological examinations, including the PSPRS with constructed subscores for “Gait and midline,” “Limb motor,” “Oculomotor,” “Bulbar,” “History,” and “Mentation,”^62^ the MMSE^63^, MoCA^20^, UPDRS-III^64^, the Clinical Dementia Rating Sum of Boxes^65^, the SEADL^66^, and the Functional Activities Questionnaire^67^.

### MRI data

MRI data were acquired at two sites, namely, the University of California, San Francisco, and the Massachusetts General Hospital, using 3-Tesla TIM Trio MRI scanners with a 12-channel head coil. DWIs were scanned with the following standardized parameters: echo-planner imaging, repetition time of 8200 ms; echo time of 86 ms; thickness of 2.2 mm; matrix of 100 × 100 (2.2 × 2.2 mm); b-values of 0 and 2000 s/mm^2^; and diffusion encoding directions of 65. The T1-weighted imaging data were collected using the sequence of three-dimensional MPRAGE with the following parameters: repetition time, 2300 ms; echo time, 2.98 ms; inversion time, 900 ms; matrix, and 160 $\times \,$240 $\times \,$256 (1 mm isotropic voxel). DWI data were acquired with relatively high *b*-values (*b* = 2000 s/mm^2^), considering that higher *b*-values might have the ability to eliminate the partial volume effect using SS3T-CSD^58,59^. Additionally, we obtained the DWIs acquired with a lower *b*-value (1000 s/mm^2^) to generate FA maps for comparison purposes. The decision to use DWI with relatively low *b*-values to obtain FA maps was based on the assumption of Gaussian diffusion in DTI^68^. The DWI with a *b*-value of 1000 s/mm^2^ was obtained using echo-planner imaging, following specific acquisition parameters: repetition time of 9200 ms; echo time of 82 ms; thickness of 2.7 mm; matrix of 128 × 128 (2.7 × 2.7 mm); *b*-values of 0 and 1000 s/mm^2^; and diffusion encoding directions of 41. Notably, DWIs in the 4RTNI and FTLDNI projects were acquired using separate single-shell schemes, each with different parameters (*b* = 1000 s/mm^2^ or *b* = 2000 s/mm^2^). Hence, not all study participants who met the inclusion criteria had DWIs acquired with a *b*-value of 1000 s/mm^2^ (only 85% in PSP, 94% in CBS, and 90% in HC).

### Diagnostic evaluation based on classical imaging findings

To assess the diagnostic ability of conventional MRI features for classifying both PSP and CBS, the neuroradiologists (K.K. and C.A.) assessed the “hummingbirds sign” in PSP and asymmetrical frontoparietal atrophy in CBS as classical MRI findings by referring to the T1-weighted images of all participants. Discrepancies in the neuroradiological assessment were resolved by consensus.

### Missing data handling

Patients with missing data at either baseline or follow-up were omitted from the correlation analysis because the longitudinal changes in the clinical indices could not be calculated.

### Imaging analysis

#### Cross-sectional FBA at baseline

The following imaging analysis pipeline was performed using the MRtrix3Tissue (http://3tissue.github.io/) based on the recommendation by the developers^18^. The pre-processing of DWI data involved denoising with Marchenko–Pastur principal component analysis^69^ and correction for Gibbs artifacts^70^, eddy current-induced and motion-induced distortion^71^, B1 field inhomogeneities^72^, and up-sampling of the resolution with cubic b-spline interpolation to 1.3-mm isotropic voxels that were more than twice the resolution to improve the image alignment^73^. The response functions corresponding to WM, gray matter, and CSF were estimated^74^, and the group-averaged response functions were generated across all participants for each whole-brain FBA. Using group-averaged response functions, the FODs for all subjects were estimated based on the SS3T-CSD algorithm^74^. Also, the sum of intensities from each tissue component was normalized toward a constant value in all voxels. The group-specific averaged FOD templates were created by iterative nonlinear registration, and the FODs of all participants were normalized to the template based on FOD^75^. Then, the fixels within all voxels were defined as peaks of FOD lobes that were specifically segmented. Finally, fixel-wise metrics (FD, log-FC, and FDC) were defined on the produced fixel grid^74^. To perform a connectivity-based fixel enhancement statistical analysis^24^, a whole-brain probabilistic tractography consisting of 20 million streamlines was estimated based on the FOD template and decreased to 2 million streamlines using the spherical deconvolution-informed filtering of tractograms algorithm^76^, which can improve the quantitative nature and reduce biases in estimations of streamlines.

#### Longitudinal FBA for PSP and CBS

To accurately normalize the WM-FODs to group-specific templates in the longitudinal FBA for patient groups, FBA processing was performed as described in a previous study, that is^77^: (1) the baseline and follow-up FODs were rigidly co-registered to the midways for all individuals; (2) the co-registered FODs were then averaged to create subject-specific FOD templates; (3) the subject-specific FOD templates were used for generating group-specific FOD templates; and (4) the baseline and 1-year follow-up FODs in the original space were subsequently normalized to a group-specific FOD template through the subject-specific templates for each individual.

#### Fixel-wise tract-specific analysis with a tract segmentation method

A fixel-wise tract-specific analysis was performed to comprehensively investigate selective WM integrity in PSP and CBS. The tract segmentation method based on TractSeg^78^ was adopted to obtain the averaged fixel-wise metrics without contamination by the crossing fibers. FOD template-based whole-brain tractograms were categorized for generating tract-specific fixel masks in a FOD template space. Subsequently, the averaged FBA parameters along the tracts at each time point were calculated and cross-sectionally and longitudinally compared across all groups. The longitudinal changes in the fixel-wise indices were defined by the formula: ${Followup}-{Baseline}$ for correlation analysis with the degree of severity based on clinical indicators. The 19 tracts were selected by considering the evidence suggesting that the misfolded tau exhibited self-propagation along axons^12^ and involvement of tau in the cerebral neocortex, basal ganglia, especially in the striatum, and SCP observed in PSP and CBD^28,79^. Finally, the following tracts were adopted: SCP, CST, rostrum, genu, rostral body, anterior midbody, posterior midbody, isthmus, and splenium parts of the CC, superior longitudinal fascicle I (SLF-I), SLF-II, SLF-III, and striato-cortical, including striato-fronto-orbital, striato-prefrontal, striato-premotor, striato-precentral, striato-postcentral, striato-parietal, and striato-occipital pathways.

#### Conventional voxel-based analysis of morphometry and diffusion tensor imaging metrics

Voxel-based analyses were performed to investigate both cross-sectional and longitudinal WM changes using VBM^80^ for WM volume and VBQ^81^ for FA. FA maps were generated using the DTIFIT tool from the FMRIB Software Library version 6.0 (FMRIB Software Library v6.0, www.fmrib.ox.ac.uk/fsl), utilizing DWI acquired with a *b* = 1000 s/mm^2^. The VBQ was also performed using FA maps which were calculated using DWI acquired with a *b*-value of 1000 s/mm^2^. VBM and VBQ were conducted using the Statistical Parametric Mapping 12 software (https://www.fil.ion.ucl.ac.uk/spm/software/spm12/). In brief, these approaches included the following processes: (1) FA maps were co-registered into the 3D T1-weighted imaging space for each subject using a boundary-based registration; (2) the 3D T1-weighted images were segmented into WM, gray matter, and CSF; (3) the WM and FA maps were normalized to the Montreal Neurological Institute/International Consortium for Brain Mapping 152 standard space using the DARTEL (Diffeomorphic Anatomical Registration and Exponentiated Lie algebra); (4) the normalized maps were resampled to a 1-mm isolated voxel, and only WM maps were modulated; and (5) a kernel of 8-mm full width at half maximum was applied only to the normalized WM maps to smoothen them.

### Statistical analysis

#### Demographic and clinical assessments

The Statistical Package for the Social Sciences for Windows, Release 25.0 (SPSS, IBM Corporation, Armonk, NY, USA), was used for all analyses. First, we performed a Kolmogorov–Smirnov test to evaluate the normality of the data. All assessments of participants at baseline were evaluated using Student’s *t*-test or the Mann–Whitney *U* test for group-wise comparisons, and ANOVA with Tukey–Kramer as a post-hoc analysis or Kruskal–Wallis test was applied to compare the three groups. For comparisons of the categorical variables, a χ2 test was used. *P*-values <0.05 were considered significant. All cross-sectional statistical analyses were examined using indices at baseline.

#### Cross-sectional and longitudinal whole-brain FBA

To assess the fixel-wise metrics (i.e., FD, log-FC, and FDC), a general linear model was used, which included fiber-specific smoothing using whole-brain tractograms and statistical inference with default parameters (C = 0.5; E = 2; H = 3; and smoothing = 10 mm full width at half maximum)^24^. The non-parametric 10,000-permutation test was performed next to assign the family-wise error-corrected *P*-value to each fixel. We performed a group comparison in fixel-wise metrics at baseline across all groups after adjusting age, sex, and intracranial volume (log-transformed intracranial volume for log-FC) measured using FreeSurfer 6.0.1 (http://surfer.nmr.mgh.harvard.edu/fswiki)^82^ as covariates. The family-wise error-corrected *P*-value <0.05 was considered significant.

#### Voxel-based analysis

The voxel-based analysis, including VBM and VBQ, was performed using an unpaired *t*-test for cross-sectional comparisons and a paired *t*-test for longitudinal changes. We adjusted age, sex, and intracranial volume in the cross-sectional analysis. The family-wise error-corrected *P*-value of <0.05 was considered significant.

#### Fixel-wise tract-specific analysis

All statistical analyses in the fixel-wise tract-specific analysis were performed with IBM SPSS Statistics version 27.0 (IBM Corporation, Armonk, NY, USA). ANOVA with Tukey–Kramer was performed to compare the mean FD, log-FC, and FDC across groups at baseline. Paired *t*-test was used for pairwise comparisons of the longitudinal changes over 1 year of fixel-wise metrics in PSP and CBS. A receiver operating characteristic analysis was performed based on classical MRI features (i.e., hummingbird sign for PSP and asymmetrical frontoparietal atrophy for CBS). Absolute inter-rater agreement and the kappa statistic (κ) were calculated to confirm the inter-rater reliability. In addition, the linear discriminant analysis classification was also performed using the mean of log-FC and FDC along all fixels overlapping the bilateral SCP defined by the TractSeg software, which differed significantly between CBS and PSP. First, the average log-FC or FDC values were computed across all fixels along both SCP sides. The log-FC or FDC values were subsequently extracted from fixel masks that encompassed fixels demonstrating statistically significant changes in the SCP. Leave-one-out cross-validation was adopted for the linear discriminant analysis, considering the relatively small sample size of the cohort in this study. The sensitivity, specificity, and the area under the curve were then calculated. Partial correlations between the longitudinal changes of fixel-wise parameters and clinical indices were calculated with Spearman rank correlations accounting for age, sex, and disease duration at baseline. Changes across time points were calculated as baseline values minus the 1-year follow-up values. Additionally, a bootstrapping procedure of 1000 samples was applied to estimate the 95% confidence interval. In all analyses, the FDR-corrected^83^ *P* < 0.05 was considered significant for multiple comparisons of the number of WM tracts. The percentage of cases included in the correlation analysis were as follows: PSPRS in 60% of PSP and 65% of CBS cases; UPDRS-III in 70% of PSP and 59% of CBS cases; MoCA in 50% of PSP and 59% of CBS cases; and MMSE in 70% of PSP and 53% of CBS cases. Additionally, we utilized a forward selection linear regression model to assess the possibility that the baseline fixel-wise metrics, which were significantly correlated with clinical assessment parameters, could predict the changes in clinical indices over 1 year in PSP and CBS.

### Reporting summary

Further information on research design is available in the Nature Research Reporting Summary linked to this article.

### Supplementary information

Supplementary materials

Reporting Summary

## Data Availability

All subjects’ imaging data and clinical information are available in the 4RTNI and FTLDNI database (http://4rtni-ftldni.ini.usc.edu/) after agreeing to the data terms.
