# Supplementary material for: Fiber-specific micro- and macroscopic white matter alterations in progressive supranuclear palsy and corticobasal syndrome
Source: NPJ Parkinsons Dis. 2023 Aug 17;9:122. doi: 10.1038/s41531-023-00565-2 (PMC10435458; doi:10.1038/s41531-023-00565-2)
Supplement: Supplementary file 1 — Supplementary materials [file 41531_2023_565_MOESM1_ESM.pdf]

Fiber-specific micro- and macroscopic white matter alterations in progressive supranuclear palsy and corticobasal syndrome

Wataru Uchida,[1] Koji Kamagata,[1*] Christina Andica,[1, 2] Kaito Takabayashi,[1] Yuya Saito,[1] Mana Owaki,[1, 3] Shohei Fujita,[1] Akifumi Hagiwara,[1] Akihiko Wada,[1] Toshiaki Akashi,[1] Katsuhiro Sano,[1] Masaaki Hori,[4] Shigeki Aoki[1, 2]

Supplementary materials

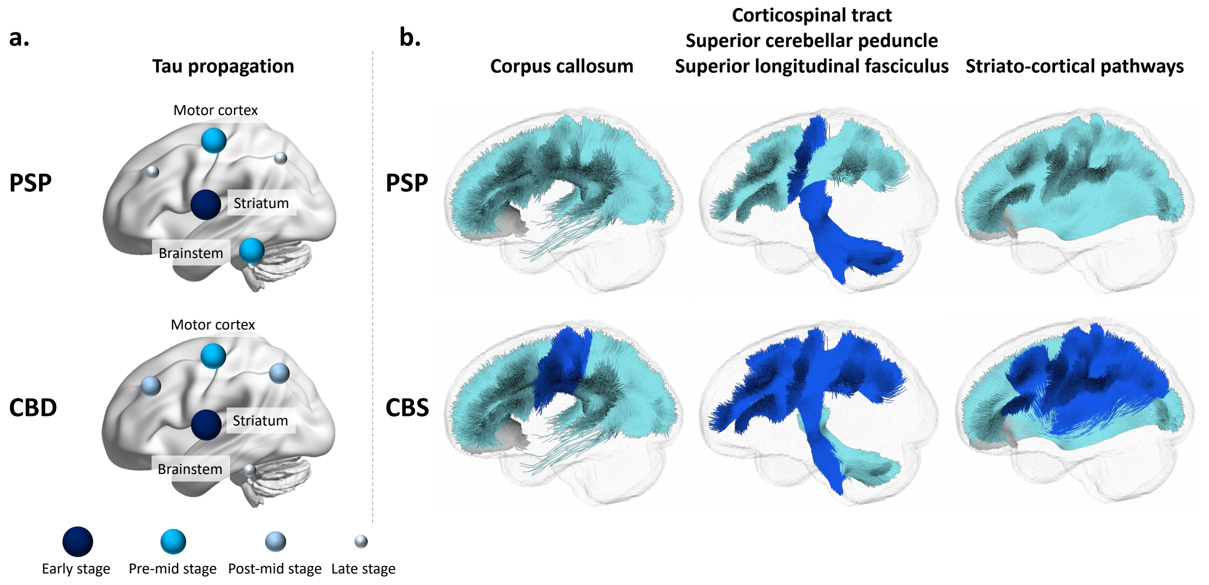

Supplementary Figure 1. Scheme of tau propagation in progressive supranuclear palsy and corticobasal degeneration and correspondence with the fixel-based analysis (FBA) results. **a** The left panel shows the propagating scheme of tau pathology in the early to late stages in progressive supranuclear palsy (PSP) and corticobasal degeneration (CBD) reported in the previous report.[52] **b** The right panel indicates the tracts that were used in the tract-specific analysis. The tracts are colored according to the significant changes in PSP and corticobasal syndrome (CBS) compared with that in healthy controls, as follows: blue, tracts significantly decreased the log-transformed fiber cross-section (log-FC) regardless of the changes of fiber density (FD) and fiber density and cross-section (FDC); light blue, tracts with no changes in log-FC and FD or FDC changed; gray, tracts showed no-significant changes in any parameters.

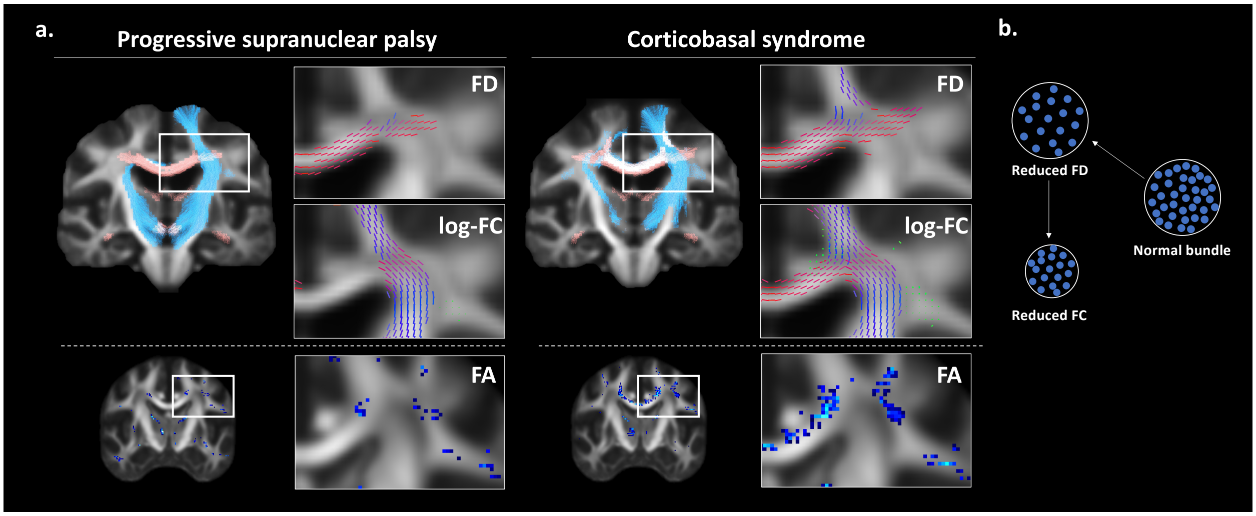

Supplementary Figure 2. The superiority of fixel-based analysis in the crossing fiber region. a The significant changes in fixel-wise metrics of fiber density (FD) and log-transformed fiber cross-section (log-FC) are shown in the upper panel (family-wise error-corrected $P < 0.05$), and voxel-wise metrics of fractional anisotropy (FA) are shown in the lower panel (uncorrected $P < 0.001$), displayed in progressive supranuclear palsy and corticobasal syndrome compared with that in healthy controls. As shown in the upper left, the whole-brain tractgrams were cropped by significantly decreased FD (orange) and log-FC (blue). The upper right panel represents the significant fixels colored by fiber direction (red, left-right; green, anterior-posterior; blue, inferior-superior) for each parameter. The lower panel indicates that the significant voxels shown in FA comparisons were scaled by t-values (range, 3 to 6). **b** Schematic of FD and log-FC reduction exhibiting changes in the fixel-wise metrics. The fiber density decreased due to neuronal loss from normal fiber bundles, followed by macroscopic atrophy.
