# Supplementary material for: Fiber-specific micro- and macroscopic white matter alterations in progressive supranuclear palsy and corticobasal syndrome
Source: NPJ Parkinsons Dis. 2023 Aug 17;9:122. doi: 10.1038/s41531-023-00565-2 (PMC10435458; doi:10.1038/s41531-023-00565-2)
Supplement: Supplementary file 2 — Reporting Summary [file 41531_2023_565_MOESM2_ESM.pdf]

nature portfolio

Corresponding author(s): Koji Kamagata

Last updated by author(s): Jul 19, 2023

Reporting Summary

Nature Portfolio wishes to improve the reproducibility of the work that we publish. This form provides structure for consistency and transparency in reporting. For further information on Nature Portfolio policies, see our Editorial Policies and the Editorial Policy Checklist.

Statistics

For all statistical analyses, confirm that the following items are present in the figure legend, table legend, main text, or Methods section.

n/a	Confirmed	
☐	☒	The exact sample size (n) for each experimental group/condition, given as a discrete number and unit of measurement
☐	☒	A statement on whether measurements were taken from distinct samples or whether the same sample was measured repeatedly
☐	☒	The statistical test(s) used AND whether they are one- or two-sided<br>*Only common tests should be described solely by name; describe more complex techniques in the Methods section.*
☐	☒	A description of all covariates tested
☐	☒	A description of any assumptions or corrections, such as tests of normality and adjustment for multiple comparisons
☐	☒	A full description of the statistical parameters including central tendency (e.g. means) or other basic estimates (e.g. regression coefficient) AND variation (e.g. standard deviation) or associated estimates of uncertainty (e.g. confidence intervals)
☐	☒	For null hypothesis testing, the test statistic (e.g. F, t, r) with confidence intervals, effect sizes, degrees of freedom and P value noted<br>*Give P values as exact values whenever suitable.*
☒	☐	For Bayesian analysis, information on the choice of priors and Markov chain Monte Carlo settings
☒	☐	For hierarchical and complex designs, identification of the appropriate level for tests and full reporting of outcomes
☒	☐	Estimates of effect sizes (e.g. Cohen's d, Pearson's r), indicating how they were calculated

Our web collection on statistics for biologists contains articles on many of the points above.

Software and code

Policy information about availability of computer code

Data collection	All subject’s imaging data and clinical information were obtained from the 4R Tauopathy Neuroimaging Initiative (4RTNI) and Neuroimaging Initiative for Frontotemporal Lobar Degeneration (FTLDNI) database after agreeing to the data terms through the website (http://4rtni-ftldni.ini.usc.edu).
Data analysis	All neuroimaging analyses were performed with neuroimaging tools including FMRIB Software Library (FSL) v6.0, MRtrix3Tissue, TractSeg, FreeSurfer, and Statistical Parametric Mapping 12 software. IBM SPSS Statistics version 27.0 was used for other statistical analyses.

For manuscripts utilizing custom algorithms or software that are central to the research but not yet described in published literature, software must be made available to editors and reviewers. We strongly encourage code deposition in a community repository (e.g. GitHub). See the Nature Portfolio guidelines for submitting code & software for further information.

Data

Policy information about availability of data

All manuscripts must include a data availability statement. This statement should provide the following information, where applicable:

- Accession codes, unique identifiers, or web links for publicly available datasets
- A description of any restrictions on data availability
- For clinical datasets or third party data, please ensure that the statement adheres to our policy

All subject's imaging data and clinical information can be available in the 4RTNI and FTLDNI database (http://4rtni-ftldni.ini.usc.edu/) after agreeing to the data terms.

Research involving human participants, their data, or biological material

Policy information about studies with human participants or human data. See also policy information about sex, gender (identity/presentation), and sexual orientation and race, ethnicity and racism.

Reporting on sex and gender	Analyses were stratified by biological sex.
Reporting on race, ethnicity, or other socially relevant groupings	Race, ethnicity and other socially relevant categories were not used in this study.
Population characteristics	Our dataset included 57 subjects consisting of 20 PSP with mean age of 69.0 at baseline and 17 CBS patients with mean age of 65.6 at baseline and 20 healthy controls with mean age of 64.7 at baseline.
Recruitment	CBS and PSP data were collected from the 4RTNI and HC data were obtained from the FTLDNI.
Ethics oversight	Ethical approval was not required because this study involves only participants from above databases previously collected and fully anonymized. The original data collection involved obtaining written informed consent from all participants in this study, and the protocol was approved by the institutional review board at all participating sites.

Note that full information on the approval of the study protocol must also be provided in the manuscript.

Field-specific reporting

Please select the one below that is the best fit for your research. If you are not sure, read the appropriate sections before making your selection.

☐ Life sciences ☒ Behavioural & social sciences ☐ Ecological, evolutionary & environmental sciences

For a reference copy of the document with all sections, see nature.com/documents/nr-reporting-summary-flat.pdf

Behavioural & social sciences study design

All studies must disclose on these points even when the disclosure is negative.

Study description	This study used retrospective design using the open-access 4RTNI and FTLDNI databases.
Research sample	The baseline and one year follow-up data, including patients with progressive supranuclear palsy (PSP) and corticobasal syndrom (CBS) from 4RTNI database and healthy controls from FTLDNI database.
Sampling strategy	We included all participants who met inclusion criteria.
Data collection	Refer to https://clinicaltrials.gov/ct2/show/NCT01804452 and https://cind.ucsf.edu/research/grants/frontotemporal-lobar-degeneration-neuroimaging-initiative-0.
Timing	Refer to https://clinicaltrials.gov/ct2/show/NCT01804452 and https://cind.ucsf.edu/research/grants/frontotemporal-lobar-degeneration-neuroimaging-initiative-0.
Data exclusions	The data of participants in our cohort who did not meet the inclusion criteria were excluded.
Non-participation	Refer to https://clinicaltrials.gov/ct2/show/NCT01804452 and https://cind.ucsf.edu/research/grants/frontotemporal-lobar-degeneration-neuroimaging-initiative-0.
Randomization	N/A

Reporting for specific materials, systems and methods

We require information from authors about some types of materials, experimental systems and methods used in many studies. Here, indicate whether each material, system or method listed is relevant to your study. If you are not sure if a list item applies to your research, read the appropriate section before selecting a response.

Materials & experimental systems

n/a	Involved in the study
☒	☐ Antibodies
☒	☐ Eukaryotic cell lines
☒	☐ Palaeontology and archaeology
☒	☐ Animals and other organisms
☒	☐ Clinical data
☒	☐ Dual use research of concern
☒	☐ Plants

Methods

n/a	Involved in the study
☒	☐ ChIP-seq
☒	☐ Flow cytometry
☐	☒ MRI-based neuroimaging

Magnetic resonance imaging

Experimental design

Design type: No task was run. We only used diffusion-weighted imaging (DWI).

Design specifications: See Acquisition below.

Behavioral performance measures: no behavioural measures were collected.

Acquisition

Imaging type(s): DWI

Field strength: 3 Tesla

Sequence & imaging parameters: 1st scan: Echo-planner imaging, repetition time, 8,200 ms; echo time, 86 ms; thickness, 2.2 mm; matrix, 100 × 100 (2.2 × 2.2 mm); b-values, 0 and 2,000 s/mm^2, and diffusion encoding directions of 65.
2nd scan: diffusion encoding directions, 65. Echo-planner imaging, repetition time, 9,200 ms; echo time, 82 ms; thickness, 2.7 mm; matrix, 128 × 128 (2.7 × 2.7 mm); b-values, 0 and 1,000 s/mm^2; and diffusion encoding directions, 41.

Area of acquisition: Whole brain DWIs were used.

Diffusion MRI: ☒ Used ☐ Not used

Parameters: 1st scan: b-values, 0 and 2,000 s/mm^2; and diffusion encoding directions, 65.
2nd scan: b-values, 0 and 1,000 s/mm^2; and diffusion encoding directions, 41.

Preprocessing

Preprocessing software: The pre-processing of DWI data involved denoising with Marchenko–Pastur principal component analysis and correction for Gibbs artifacts, eddy current-induced and motion-induced distortion, B1 field inhomogeneities, and up-sampling of the resolution with cubic b-spline interpolation to 1.3-mm isotropic voxels using MRtrix3Tissue.

Normalization: The response functions corresponding to white matter, gray matter, and CSF were estimated, and the group-averaged response functions were generated across all participants for each whole-brain FBA. Using group-averaged response functions, the fiber orientation distributions (FODs) for all subjects were estimated based on the single-shell three-tissue constrained spherical deconvolution (SS3T-CSD) algorithm. Also, the sum of intensities from each tissue component was normalized toward a constant value in all voxels. The group-specific averaged FOD templates were created by iterative nonlinear registration, and the FODs of all participants were normalized to the template based on FOD.

Normalization template: All DWI in subjects space were normalized to group-specific template space.

Noise and artifact removal: Denoising with Marchenko–Pastur principal component analysis and correction for Gibbs artifacts, eddy current-induced and motion-induced distortion, B1 field inhomogeneities were performed for all DWIs.

Volume censoring: No volume censoring was performed.

Statistical modeling & inference

Model type and settings

Fiber density (FD), fiber cross-section (FC), and fiber density and cross-section (FDC) were obtained for fixel-wise analyses. Fractional anisotropy maps were then calculated for voxel-based quantification

Effect(s) tested

Group differences of FBA parameters including FD, log-FC, and FDC at baseline were assessed. Longitudinally, FBA metrics were also evaluated.

Specify type of analysis: ☐ Whole brain ☐ ROI-based ☒ Both

Anatomical location(s)

The tract segmentation method based on TractSeg was adopted to obtain the averaged fixel-wise metrics without contamination by the crossing fibers. Finally, the following tracts were adopted: SCP, CST, rostrum, genu, rostral body, anterior midbody, posterior midbody, isthmus, and splenium parts of the CC, superior longitudinal fascicle I (SLF-I), SLF-II, SLF-III, and striato-cortical, including striato-fronto-orbital, striato-prefrontal, striato-premotor, striato-precentral, striato-postcentral, striato-parietal, and striato-occipital pathways.

Statistic type for inference

(See Eklund et al. 2016)

Whole-brain FBA: To assess the fixel-wise metrics, a general linear model was used, which included fiber-specific smoothing using whole-brain tractograms and statistical inference with default parameters (C=0.5; E=2; H=3; and smoothing=10 mm full width at half maximum).We performed a group comparison in fixel-wise metrics at baseline across all groups after adjusting age, sex, and intracranial volume (log-transformed intracranial volume for log-FC) as covariates.

Whole-brain voxel-based anlaysis: VBM and VBQ, were performed using an un-paired t-test for cross-sectional comparisons and a paired t-test for longitudinal changes. We adjusted age, sex, and intracranial volume in the cross-sectional analysis.

Fixel-wise tract-specific anlaysis: ANOVA with Tukey–Kramer was performed to compare the mean FD, log-FC, and FDC across groups at baseline. Paired-t test was used for pairwise comparisons of the longitudinal changes over one year of fixel-wise metrics in PSP and CBS.

Correction

Whole-brain FBA: The non-parametric 10,000-permutation test was performed next to assign the family-wise error corrected P-value to each fixel. The family-wise error corrected P-value < 0.05 was considered significant.

Whole-brain voxel-based anlaysis: The family-wise error corrected P-value of < 0.05 was considered significant.

Models & analysis

n/a	Involved in the study
☒	☐ Functional and/or effective connectivity
☒	☐ Graph analysis
☐	☒ Multivariate modeling or predictive analysis

Multivariate modeling and predictive analysis

A forward selection in the linear regression model was used to test the possibility that fixel-wise metrics could predict future dysfunction in PSP and CBS.
